# Efficacy, safety and feasibility of fosaprepitant for the prevention of chemotherapy-induced nausea and vomiting in pediatric patients receiving moderately and highly emetogenic chemotherapy – results of a non-interventional observation study

**DOI:** 10.1186/s12885-019-6252-6

**Published:** 2019-11-15

**Authors:** Semjon Willier, Karin Melanie Cabanillas Stanchi, Martina von Have, Vera Binder, Franziska Blaeschke, Judith Feucht, Tobias Feuchtinger, Michaela Döring

**Affiliations:** 1 Dr.-von-Hauner’sches Kinderspital, Paediatric Haematology, Oncology and Stem Cell Transplantation, Ludwig-Maximilians-University München, 80337 Munich, Germany; 2 Department I – General Paediatrics, Haematology/Oncology, University Children’s Hospital Tübingen, Hoppe-Seyler-Str. 1, 72076 Tübingen, Germany

**Keywords:** Fosaprepitant, Aprepitant, Pediatric patients, Emetogenic chemotherapy, Chemotherapy-induced nausea and vomiting, Ondansetron, Antiemetic prophylaxis, ALL, Non-interventional observation study

## Abstract

**Background:**

Chemotherapy-induced nausea and vomiting (CINV) belong among the most burdensome side effects in hemato-oncology. Mostly, a combination of ondansetron and dexamethasone is used as antiemetic prophylaxis in pediatric patients undergoing emetogenic chemotherapy. However, dexamethasone is prohibited in different pediatric chemotherapy protocols. Currently, data on the use of ondansetron with the new antiemetic agent fosaprepitant without dexamethasone is not available for pediatric patients.

**Methods:**

In this non-interventional observation study, 79 pediatric patients with a median age of 8.0 years (range 0.5–17.9 years) who received a CINV prophylaxis regimen with either fosaprepitant (4 mg/kg; maximum 150 mg) and ondansetron (as *24-h* continuous *infusion*) (*n* = 40; fosaprepitant group/FG) or ondansetron only (*n* = 39; control group/CG) during moderately or highly emetogenic chemotherapy were analyzed. The groups were analyzed and compared for frequency of vomiting, administered doses of on-demand antiemetic dimenhydrinate and adverse events during the acute (0-24 h after chemotherapy administration) and delayed (> 24 h–120 h) CINV phases.

**Results:**

A total of 112 and 116 chemotherapy blocks were analyzed in the fosaprepitant and the control group, respectively. The emetogenic potential of the administered chemotherapy did not significantly differ (*p* = 0.8812) between the two cohorts. In the acute CINV phase, the percentage of patients experiencing vomiting (*n* = 26 patients) and the vomiting events were significantly higher (*p* = 0.0005 and *p* < 0.0001, respectively) in the CG (*n* = 26 patients (66.7%); 88 events) compared with the FG (*n* = 10 patients (25.0%); 37 events). In the delayed CINV phase, the percentage of patients experiencing vomiting and the vomiting events were also significantly higher (*p* = 0.0017 and *p* < 0.0001, respectively) in the CG (*n* = 31 patients (79.5%); 164 events) compared with the FG (*n* = 17 patients (42.5%); 103 events). Additionally, significantly more dimenhydrinate doses were administered in the CG compared with the FG patients (*n* = 322/*n* = 198; *p* < 0.0001). The occurrence of adverse events did not significantly differ between the two groups (*p* > 0.05).

**Conclusion:**

Fosaprepitant (4.0 mg/kg) in addition to ondansetron, without application of dexamethasone, was well tolerated, safe, effective and superior to ondansetron only as CINV prophylaxis in pediatric patients during moderately and highly emetogenic chemotherapy.

## Background

Chemotherapy-induced nausea and vomiting (CINV) is the most common and the most burdensome side effect associated with anti-cancer treatment. Especially in pediatric patients, this chemotherapy-related adverse event poses a significant impact on the quality of life [1].

During emetogenic chemotherapy, receptors of specific regions of the vomiting center of the brain may be activated. These receptors are usually bound by three different neurotransmitters: serotonin (5-hydroxytryptamine-3 receptor (5-HT_3_R)), substance P (neurokinin-1 receptor (NK_1_R) and dopamine (D_2_ receptor), inducing peripherally- or centrally-caused nausea and vomiting. CINV may occur within the first 24 h (acute phase) or 24 to 120 h (delayed phase) after the administration of chemotherapy [1].

Fosaprepitant is a water-soluble prodrug that is converted to aprepitant, which selectively antagonizes the NK_1_R. In several clinical trials, aprepitant and fosaprepitant have proven effective in both the acute and delayed phases of CINV in adult and pediatric patients [2–4]. Compared with aprepitant formulations (capsules, suspension), fosaprepitant can be administered intravenously (IV) due to its hydrophilic qualities. Fosaprepitant is administered once prior to chemotherapy and every 5 days thereafter, compared with oral administration of aprepitant on three consecutive days starting on the first day of chemotherapy administration [5]. These advantages of the IV formulation hold strong importance especially in patients who are unable or unwilling to take oral formulations due to their low age or during mucositis.

A recently-published placebo-controlled trial has shown favorable results of a CINV prophylaxis regimen with fosaprepitant, ondansetron and dexamethasone in pediatric patients under 12 years of age receiving moderately or highly emetogenic chemotherapy for the treatment of hematologic and oncologic malignancies [4].

The current MASCC/ESMO (Multinational Association of Supportive Care in Cancer/European Society for Medical Oncology) guidelines recommend an antiemetic prophylaxis regimen with a 5-HT_3_R antagonist plus the NK_1_R antagonist aprepitant plus dexamethasone for pediatric patients during moderately to highly emetogenic chemotherapy [5]. However, due to concerns regarding the immunosuppressive effects increasing the risk of infection (e.g. fungal infections) and interferences with the distribution of chemotherapy through the blood-brain barrier and apoptotic processes, dexamethasone is prohibited in several pediatric chemotherapy protocols [5, 6].

Until October 2015, the standard antiemetic prophylaxis regimen of the children’s hospital where the study was conducted included ondansetron only as a 24-h continuous infusion and additional pro re nata (PRN) medication with dimenhydrinate. Due to excellent results regarding the efficacy and safety of CINV prophylaxis with fosaprepitant in adult patients [2] and initial studies in pediatric patients [7], the CINV prophylaxis strategy for pediatric patients receiving moderately to highly emetogenic chemotherapy was gradually expanded with single-dose fosaprepitant. Initially, older children ≥12 years of age received fosaprepitant. After a good tolerability was seen in these patients, younger children ≥6 and then ≥2 years and ≥ 0.5 years of age were gradually changed to a fosaprepitant-based regimen. Due to the very good clinical experience with fosaprepitant, the standard CINV prophylaxis regimen for these patients was changed to fosaprepitant plus ondansetron in 2015. In April 2018, the US Food and Drug Administration approved fosaprepitant as CINV prophylaxis and therapy in pediatric patients between 0.5 and 17 years of age [8].

The primary objective of this non-interventional observation study was to evaluate the efficacy, safety and feasibility of an antiemetic prophylaxis regimen with single-dose fosaprepitant plus ondansetron without dexamethasone in pediatric hemato-oncological patients in routine clinical practice in comparison with a standard regimen with ondansetron only for the prevention of CINV caused by moderately and highly emetogenic chemotherapy.

## Methods

### Study design

This non-interventional observational study analyzed data of pediatric patients between 0.5 and 17 years of age who were treated at the Department of Pediatric Hematology and Oncology at the Dr. von Hauner Children’s Hospital, Germany between November 2015 and August 2016 receiving one or more chemotherapy courses for the treatment of hemato-oncological diseases according to pediatric oncology protocols.

The EP of the administered chemotherapeutic agent (in % frequency of emesis in absence of prophylaxis) was defined as: minimal, stage 1 (< 10%) | low, stage 2 (10 - < 30%) | moderate, stage 3 (30–90%) | high, stage 4 (> 90%) [9]. The EP of each chemotherapy course was defined by the administered agent with the highest EP (≥3; compare Table 3).

Inclusion criteria were age at the time of chemotherapy administration between 0.5–17 years, administration of chemotherapy with a moderately or highly emetogenic potential (stage 3–4) during an in-patient stay, antiemetic prophylaxis with ondansetron only or ondansetron plus single-dose fosaprepitant.

Exclusion criteria were vomiting in the 24 h prior to the start of emetogenic chemotherapy, (additional) medication with aprepitant, granisetron, or dexamethasone, allergy to NK_1_ or 5-HT_3_-antagonists, congestive heart failure, abnormal liver (AST and ALT > 2.5-fold of the upper normal limit) or kidney (serum creatinine > 2.5-fold of the upper normal limit) function in the 24 h prior to the start of emetogenic chemotherapy, and scheduled hematopoietic stem cell transplantation.

All patients who met the inclusion criteria and received single-dose fosaprepitant and 24-h continuous infusion with ondansetron between November 2015 and August 2016 were consecutively enrolled in the fosaprepitant group (FG; *n* = 40). All patients who met the inclusion criteria and received CINV prophylaxis with ondansetron only between November 2014 and October 2015 were consecutively enrolled in the control group (CG; *n* = 39).

The analysis period with fosaprepitant and/or ondansetron included the time between the start of antiemetic prophylaxis until 120 h after starting the first moderately or highly emetogenic agent of each chemotherapy course.

The acute CINV phase of each chemotherapy course was defined as the first 24 h after the first application of a moderately or highly emetogenic agent. The delayed CINV phase was defined as the subsequent 96 h (> 24–120 h after administration of the first moderately or highly emetogenic agent).

Primary endpoints were the assessment of the efficacy (frequency of vomiting, number of patients who experienced vomiting; frequency of administration of antiemetic PRN medication dimenhydrinate) and safety (clinical and laboratory-chemical adverse events) of the prophylaxis regimens.

### Drug administration

Ondansetron intravenous administration through a central venous catheter was started at least 30 min prior to the start of the first moderately or highly chemotherapeutic agent of each chemotherapy course at dosages of 8 mg per 24 h in patients ≤15 kg bodyweight, 16 mg per 24 h in patients of > 15–30 kg bodyweight, 24 mg per 24 h in patients of > 30–45 kg bodyweight, and a maximum dose of 32 mg per 24 h in patients with a bodyweight of > 45 kg as a 24-h continuous infusion over the whole time of intravenous chemotherapy administration until 24 h after the administration of the last agent of the respective course. Subsequently, the patients were supplied with ondansetron tablets or orally disintegrating tablets (2 × 2–8 mg per day; adapted to the body surface area).

Fosaprepitant was started at least 1 h prior to the start of the first moderately or highly chemotherapeutic agent of each chemotherapy course as a single dose of 4.0 mg per kg bodyweight (maximum 150 mg) as intravenous infusion through a central venous catheter over 30 min.

Dimenhydrinate was provided as PRN medication through a central venous catheter infusion (1.0 mg per kg BW three times per day, max. 3 × 62 mg short infusion).

Breakthrough CINV was treated with dimenhydrinate (dosage 0.1 mg per kg BW per day as 24 h infusion (max. 0.2 mg/kg per day BW)).

### Assessment of safety and tolerance

The toxicity and adverse events grading of this analysis is based on the current United States National Cancer Institute’s Common Terminology Criteria for Adverse Events [10]. Analyses of liver parameters, kidney parameters and electrolytes were performed on the day of in-patient admission before the first chemotherapy course and antiemetic prophylaxis (baseline), at least every other day during antiemetic prophylaxis during the in-patient stay (maximum or minimum), as well as on the day of clinical discharge (end), i.e. during the whole analysis period.

Liver parameters included alanine aminotransferase (ALT, normal range ≤ 39 U/L) and aspartate aminotransferase (AST, normal range ≤ 59 U/L), and the cholestasis parameter total bilirubin (normal range ≤ 1.1 mg/dL). Kidney parameters included serum creatinine (normal range ≤ 0.7 mg/dL) and urea (normal range ≤ 46 mg/dL). Electrolytes included potassium (normal range ≥ 3.4 mmol/L - 4.9 mmol/L), sodium (normal range 134 mmol/L - 145 mmol/L), and calcium (≥2.0 mmol/L - 2.6 mmol/L). Clinically-relevant elevations of > 1.5 and > 2.5 times the normal values of hepatic and kidney parameters, and decreases of potassium values < 3.4 mmol/L or < 2.4 mmol/L, sodium values < 134 mmol/L and calcium values < 2.0 mmol/L were assessed. Maximum (ALT, AST, total bilirubin, creatinine, and urea) or minimum (potassium, sodium, calcium) values during the analysis period were used for comparisons with baseline values. Clinical potentially drug-related adverse events were analyzed during the application of fosaprepitant and compared between the two groups.

### Assessment of efficacy

All patients were primarily monitored for the efficacy of the antiemetic prophylaxis regimen during the acute and delayed CINV phase of moderately and highly emetogenic chemotherapy courses. The vomiting frequency of the patients during the acute and delayed CINV phases and the number of administered doses of dimenhydrinate during all chemotherapy courses of both study cohorts was used as the measure for the efficacy analyses of both prophylaxis regimens. The relative number of patients experiencing vomiting and receiving dimenhydrinate was analyzed. The assessed parameters were analyzed and compared between the two study cohorts.

### Statistical analysis

Chi-square-tests (with Yates’ continuity correction) and Fisher’s exact tests were used for 2-sample tests for equality of proportions and applied to the frequencies of clinical parameters in the two treatment groups (FG and CG). In addition, the package rateratio.test of R was used to compare the frequency of the vomiting events between FG and CG [11].

The statistical comparison of the differences between the results and the normal range values for the liver and kidney parameters, as well as electrolytes, was performed by one-sample t-tests or one sample Wilcoxon signed rank tests (depending on the results of the Shapiro-Wilk normality test), taking into account the 95% confidence intervals (CI).

The inferential statistical analysis between the baseline values, as well as the maximum and minimum values, was performed with the Wilcoxon matched pairs signed rank test. Differences were only considered to be significant if they were clinically relevant, i.e. significantly below (sodium, calcium and potassium) the reference values or above them (all other parameters).

Graphs and statistical tests were created with GraphPad Prism for Windows, version 7 (GraphPad Software Inc., La Jolla, CA, USA), or with R (The R Foundation for Statistical Computing, Institute for Statistics and Mathematics, Wirtschaftsuniversität Wien, Austria). *P*-values of *p* < 0.05 (*), *p* < 0.01(**), *p* < 0.001 (***), and *p* < 0.0001 (****) were defined as statistically significant and are illustrated in the bar charts.

## Results

### Patient characteristics

A total of 79 pediatric patients were enrolled in this analysis. The median age at the first analyzed chemotherapy cycle was 8.0 years (range 0.5–17.9 years) in all patients, 7.4 years (range 0.5–17.9 years) in the fosaprepitant group, and 8.3 years (range 0.6 year – 17.6 years) in the control group. A significant difference regarding age and gender could not be detected between the groups (Table 1). Of the 79 patients, 40 (50.6%) received an antiemetic prophylaxis regimen with fosaprepitant and ondansetron and 39 (49.4%) received ondansetron only, respectively.

**Table 1:** Patient Characteristics

Patient Characteristics	Fosaprepitant group	Control group	*p*-value
	*N* = 40	*N* = 39	
	*n* (%)	*n* (%)	
Age			
< 2 years	5 (12.5)	5 (12.8)	0.7676
2–6 years	11 (27.5)	14 (35.9)	0.5752
7–12 years	11 (27.5)	8 (20.5)	0.6432
13–17 years	13 (32.5)	12 (30.8)	0.939
Sex			
Male	25 (62.5)	22 (56.4)	0.7474
Female	15 (37.5)	17 (43.6)	
Diagnosis			
ALL	10 (25.0)	8 (20.5)	0.8359
AML	0 (0.0)	1 (2.6)	0.4937
ALL relapse	2 (5.0)	2 (5.1)	> 0.9999
Astrocytoma	1 (2.5)	1 (2.6)	> 0.9999
Non-Hodgkin Lymphoma	4 (10.0)	5 (12.8)	0.737
Ependymoma	1 (2.5)	1 (2.6)	> 0.9999
Ewing’s sarcoma	4 (10.0)	4 (10.3)	> 0.9999
Germinoma	1 (2.5)	1 (2.6)	> 0.9999
Hepatoblastoma	3 (7.5)	2 (5.1)	> 0.9999
Hodgkin’s Lymphoma	2 (5.0)	1 (2.6)	> 0.9999
Medulloblastoma	2 (5.0)	1 (2.6)	> 0.9999
MDS	1 (2.5)	0 (0.0)	> 0.9999
Nephroblastoma	0 (0.0)	1 (2.6)	0.4937
Neuroblastoma	3 (7.5)	3 (7.7)	> 0.9999
Osteosarcoma	1 (2.5)	2 (5.1)	> 0.9999
Renal cell carcinoma	0 (0.0)	1 (2.6)	0.4937
Rhabdomyosarcoma	4 (10.0)	2 (5.1)	0.6752
T cell lymphoma	1 (2.5)	3 (7.7)	0.3589

A statistically significant difference of the patient characteristics between the two groups could not be detected (*p* > 0.05; Chi-square test with Yate’s correction or Fisher’s exact test)

*Abbreviations*: *ALL* acute lymphoblastic leukemia, *AML* acute myeloid leukemia, *N* total number of patients per cohort, *n* sample size, *MDS* myelodysplastic syndromes

### Analysis period

The median period of analysis was 6 days (range 5–9 days) in both the fosaprepitant and the control group. In the fosaprepitant group, a total of 112 chemotherapy courses were administered, of which 84 courses (75.0%) included moderately emetogenic agents and 28 courses (25.0%) included highly emetogenic agents. In the control group, a total of 116 chemotherapy courses were administered, of which 89 courses (76.7%) included moderately emetogenic agents and 27 courses (23.3%) included highly emetogenic agents. The EPs of the administered chemotherapy courses did not significantly differ between the two groups (*p* = 0.8812; Table 2). A median of 3 (range 3–4) chemotherapy courses were analyzed per patient in the fosaprepitant group and the control group. Thirty-six of the 40 patients of the fosaprepitant group (90.0%) and 37 of the 39 patients of the control group (94.9%) were observed during more than one moderately or highly emetogenic chemotherapy course (total: 92.4% of the patients). None of the patients received dexamethasone.

**Table 2:** Chemotherapy

	Dosage	EP	Fosaprepitant group	Control group	*p*-value
			*N* = 112 courses	*N* = 116 courses	
			*n* (%)	*n* (%)	
Agent					
Carboplatin		4	8 (7.1)	8 (6.9)	> 0.9999
Cisplatin		4	5 (4.5)	8 (6.9)	0.5704
Clofarabine		3	0 (0.0)	1 (0.9)	> 0.9999
Cyclophosphamide	≥1 g/m^2^	4	2 (1.8)	2 (1.7)	> 0.9999
Cyclophosphamide	< 1 g/m^2^	3	19 (17.0)	18 (15.5)	0.9072
Cytarabine	> 200 mg/m^2^ to < 3 g/ m^2^	3	16 (14.3)	11 (9.5)	0.3591
Dacarbazine		4	3 (2.7)	2 (1.7)	0.6793
Dactinomycin		4	9 (8.0)	7 (6.0)	0.6112
Daunorubicin		3	4 (3.6)	10 (8.6)	0.1669
Doxorubicin		3	16 (14.3)	10 (8.6)	0.2555
Epirubicin		3	2 (1.8)	0 (0.0)	0.2402
Etoposide		3	28 (25.0)	25 (21.6)	0.6459
Idarubicin		3	1 (0.9)	0 (0.0)	0.4912
Ifosfamide		3	17 (15.2)	12 (10.3)	0.3701
Irinotecan		3	0 (0.0)	2 (1.7)	0.498
Melphalan	> 50 mg/m^2^	3	1 (0.9)	1 (0.9)	1.0000
Methotrexate	≥250 mg to < 12 g/m^2^	3	20 (17.9)	15 (12.9)	0.3965
Temozolomide		3	0 (0.0)	1 (0.9)	> 0.9999
Thiotepa	≥300 mg/m^2^	4	1 (0.9)	0 (0.0)	0.4912
EP (CINV risk)					
3 (> 30–90%)			84 (75.0)	89 (76.7)	0.8812
4 (> 90%)			28 (25.0)	27 (23.3)	

The table shows the emetogenic potential (EP) of the administered chemotherapeutic agents in the fosaprepitant and the control group. EP was defined by the emetic risk (in % frequency of emesis in absence of prophylaxis): minimal, stage 1 (< 10%) | low, stage 2 (10 - < 30%) | moderate, stage 3 (30–90%) | high, stage 4 (> 90%) [9]

*Abbreviations*: *CINV* chemotherapy-induced nausea and vomiting, *EP* emetogenic potential, *N* total number of administered chemotherapy courses, *n* sample size. The distribution of administered chemotherapeutic agents and the highest emetogenic potential of each chemotherapy course was not significantly different in both cohorts (*p* > 0.05; Chi-square test with Yate’s correction or Fisher’s exact test)

### Efficacy analysis

**Fig. 1:**
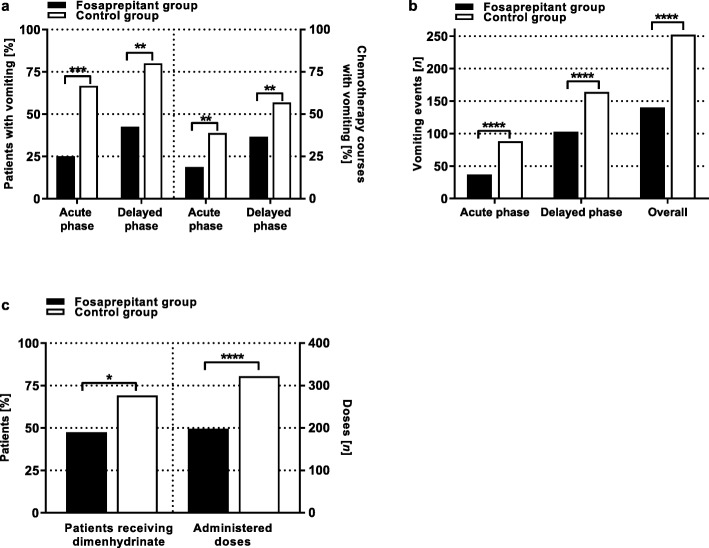
Efficacy. The graph displays analyses of patients during moderately and highly emetogenic chemotherapy and CINV prophylaxis with ondansetron only (control group; 39 patients/116 chemotherapy courses; white bars) or with a combination of ondansetron and fosaprepitant (fosaprepitant group; 40 patients/112 chemotherapy courses; black bars). a. The percentage of patients experiencing vomiting was significantly higher in the control group during both the acute (*n* = 10; 25.0% vs. *n* = 26; 66.7%; *p* = 0.0005) and the delayed (*n* = 17; 42.5% vs. *n* = 31; 79.5%); *p* = 0.0017) CINV phase. Likewise, the number of chemotherapy courses in which vomiting occurred was significantly higher in the control group during both the acute (*n* = 45; 38.8% vs. *n* = 21; 18.8%; *p* = 0.0014) and the delayed (*n* = 66; 56.9% vs. *n* = 41; 36.6%: *p* = 0.0033) CINV phase b. The total number of vomiting events during all chemotherapy courses of the respective cohort and both CINV phases was significantly higher in the control group when compared to the fosaprepitant group during the acute (88 vs. 37 events; *p* < 0.0001) and the delayed CINV phase (164 vs. 103; *p* < 0.0001), and during both CINV phases (252 vs. 140 events, *p* = 0.0001). c. Significantly (*p* = 0.04884) fewer patients of the fosaprepitant group needed PRN medication with dimenhydrinate during both CINV phases compared with the control group (47.5%, *n* = 19 vs. 69.2%, *n* = 27). 1.6-fold fewer doses of dimenhydrinate were administered in the fosaprepitant group when compared to the control group during both CINV phases (198 vs. 322; *p* < 0.0001). Symbols indicate *: *p* < 0.05 | **: *p* < 0.01 | ***: *p* < 0.001 | ****: *p* < 0.0001

All 79 of the patients were included in the efficacy analysis. The relative number of patients experiencing vomiting during the acute and the delayed CINV phase during all 112 (FG) and 116 (CG) chemotherapy courses was significantly lower (acute phase: *p* = 0.0005 | delayed phase: *p* = 0.0017) when receiving antiemetic prophylaxis with fosaprepitant and ondansetron (*n* = 10; 25.0% and *n* = 17; 42.5% respectively) compared with the patients receiving ondansetron only (*n* = 26/66.7% and *n* = 31/79.5%, respectively) (Fig. 1). Likewise, the relative number of chemotherapy courses, in which vomiting occurred was significantly higher in the control group when compared to the fosaprepitant group in both the acute CINV phase (CG: 45 of 116 courses (38.8%) vs. FG: 21 of 112 courses (18.8%); *p* = 0.0014) and the delayed CINV phase (CG: 66 of 116 courses (56.9%) vs. FG: 41 of 112 courses (36.6%); *p* = 0.0033).

The vomiting frequency was significantly lower in patients with fosaprepitant and ondansetron prophylaxis with 37 events in the acute phase and 103 events in the delayed phase as opposed to 88 events in the acute phase (*p* < 0.0001) and 164 events in the delayed phase (*p* < 0.0001) for the control group. In the delayed phase, 62 of 103 vomiting events (60.2%) of the fosaprepitant group and 105 of 164 vomiting events (64.0%) occurred within the first 48 h of the delayed phase (> 24–72 h after chemotherapy administration).

A median of 3 (range 3–4) chemotherapy courses were analyzed per patient in the fosaprepitant group and the control group. Analyzing the courses in which vomiting occurred in the fosaprepitant, a median of two vomiting events (range 1–3) occurred during the acute and a median of three events (range 1–6) during the delayed CINV phase. In the control group, a median of two events (range 1–7) occurred in the acute and a median of four (range 1–21) in the delayed CINV phase.

Analyzing repeated cycles per patient in the fosaprepitant group, a median of two vomiting events (range 1–6) were registered in the first course administered to a patient. In the second course, a median of two (range 1–4), in the third course a median of two (range 1–6) and in the fourth course a median of two (range 1–5) vomiting events were registered. In the control group, a median of three vomiting events occurred in the first course (range 1–14), a median of two (range 1–21) in the second course, and median of three (range 2–8) in the third course and a median of three events (range 2–11) in the fourth course. In conclusion, vomiting frequencies did not increase or decrease within repeated chemotherapy courses in the same patients in both the fosaprepitant and the control group.

Overall, during both CINV phases (0-120 h after chemotherapy administration), 140 vomiting events during 112 chemotherapy courses were observed in the fosaprepitant group in comparison to 252 events during 116 chemotherapy courses in the control group (significantly different; *p* < 0.0001).

This effect could also be seen in the administered doses of additional PRN medication with dimenhydrinate during both CINV phases. Thus, the number of patients receiving dimenhydrinate during both CINV phases was significantly lower (*p* = 0.04884) in the fosaprepitant group (*n* = 19; 47.5%) compared with the control group (*n* = 27; 69.2%). Likewise, the number of chemotherapy courses during which dimenhydrinate was administered during both CINV phases was significantly higher (*p* = 0.0001) in the control group (*n* = 59; 50.9%) compared with the fosaprepitant group (*n* = 28; 25.0%). Among those courses, in which dimenhydrinate was administered, a median of 2 doses (range 1–21 doses; mean 7.1 ± 6.4 doses) were administered in the control group and a median of 2 doses (range 1–12 doses; mean 3.3 ± 3.0 doses) were administered in the fosaprepitant group during both CINV phases. The total number of administered doses of dimenhydrinate during both CINV phases was significantly smaller (*p* < 0.0001) during prophylaxis with fosaprepitant and ondansetron (*n* = 198) compared with ondansetron only (*n* = 322) (Fig. 1c).

### Safety and tolerance

None of the 79 enrolled patients died during the analysis period. A discontinuation of the antiemetic medication was not indicated for any patient in either of the two groups.

During the analysis period, a significant increase of the hepatic parameter ALT could not be observed (*p* > 0.05) in either the control group (baseline: median 26 U/L | mean 30 ± 16.3 U/L | range 8–80 U/L; versus maximum: median 44 U/L | mean 52 ± 42.5 U/L | range 10–176 U/L) or the fosaprepitant group (baseline: median 21 U/L | mean 26 ± 16.9 U/L | range 5–80 U/L; versus maximum: median 34 U/L | mean 50 ± 51.3 U/L | range 4–213 U/L). Likewise, a significant increase of the hepatic parameter AST between baseline values and maximum values could not be observed (*p* > 0.05) in either the control group (baseline: median 34 U/L | mean 37 ± 13.8 U/L | range 18–79 U/L; versus maximum: median 45 U/L | mean 55 ± 40.3 U/L | range 11–180 U/L) or the fosaprepitant group (baseline: median 28 U/L | mean 32 ± 14.5 U/L | range 8–78 U/L; versus maximum: median 36 U/L | mean 53 ± 42.1 U/L | range 12–187 U/L).

**Fig. 2:**
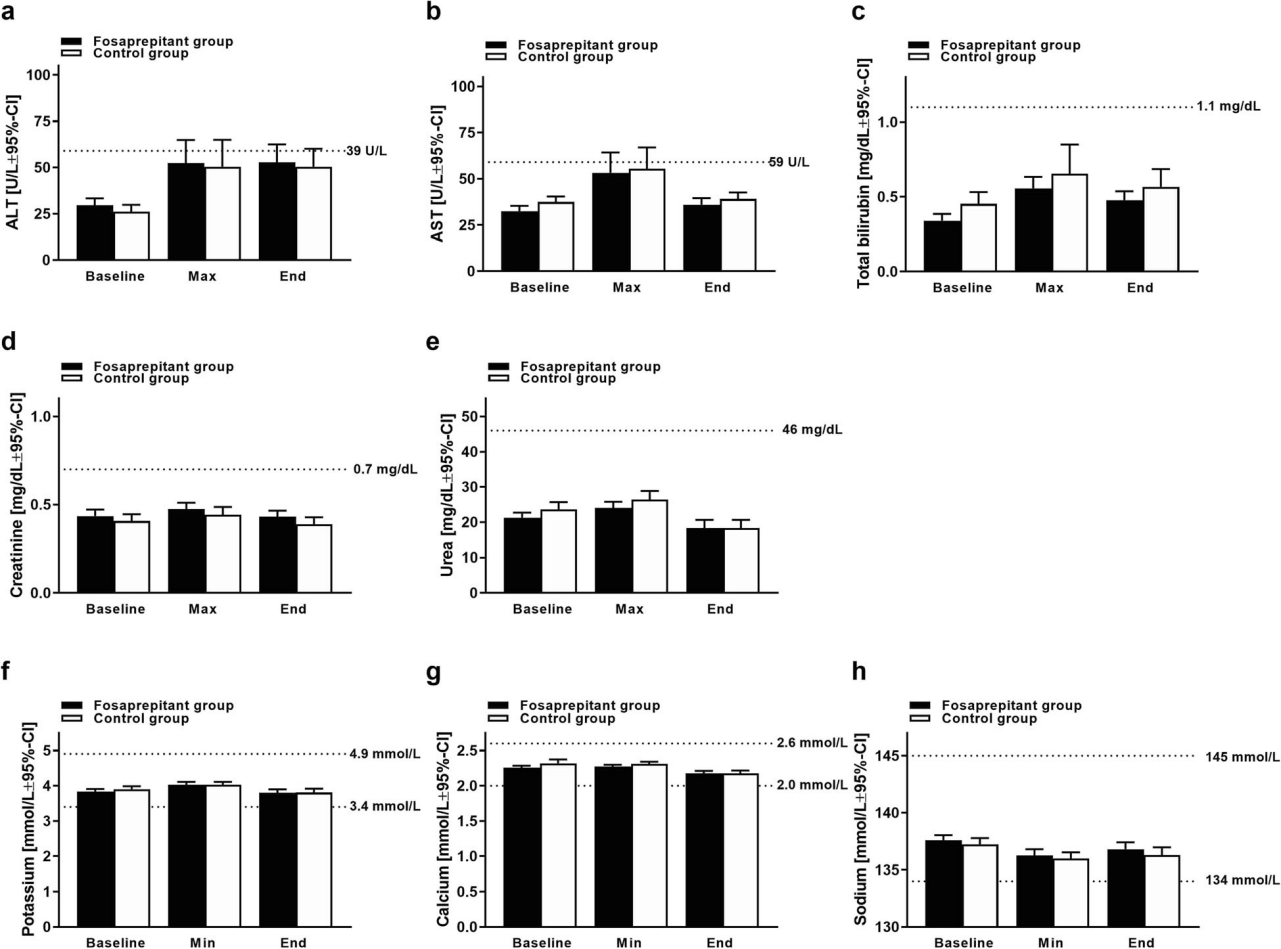
Safety. The graph displays median values + 95% confidence interval (CI) of transaminases ALT (**a**) and AST (**b**), total bilirubin (**c**), creatinine (**d**), urea (**e**), potassium (**f**), calcium (**g**) and sodium (**h**) on the day before the start of emetogenic chemotherapy (Baseline), maximum/minimum values during (Max/Min) and at the end (End) of the analysis period. Normal values are indicated by dotted lines. Mean and median values of the analyzed parameters did not increase or decrease beyond the normal values. Statistical significance was tested by the Wilcoxon matched-pairs signed-rank test

Increases of ALT and AST > 2.5 times the normal value occurred in both the fosaprepitant (6.3 and 0.9% of the patients, respectively) and the control group (6.9 and 2.6% of the patients, respectively), but were not significantly different (*p* > 0.5). Most probably, these increases are associated with the administered chemotherapy and not caused by the antiemetic medication with fosaprepitant or ondansetron (Table 3). All other analyzed laboratory parameters (total bilirubin, creatinine, urea, potassium, calcium and sodium) did not significantly or relevantly change during the observation period in either the control group or the fosaprepitant group (Fig. 2; Table 3).

**Table 3:** Adverse reactions

	Fosaprepitant group	Control group	*p*-value
	*N* = 112 courses	*N* = 116 courses	
	*n* (%)	*n* (%)	
Laboratory markers			
Increase ALT (n.v. ≤ 39 U/L)			
≥ 1.5 x normal value	7 (6.3)	6 (5.2)	0.9481
≥ 2.5 x normal value	7 (6.3)	8 (6.9)	0.8439
Increase AST (n.v. ≤ 59 U/L)			
≥ 1.5 x normal value	7 (6.3)	5 (4.3)	0.7195
≥ 2.5 x normal value	1 (0.9)	3 (2.6)	0.6390
Increase total bilirubin (n.v. ≤ 1.1 mg/dL)			
≥ 1.5 x normal value	1 (0.9)	2 (1.7)	> 0.9999
≥ 2.5 x normal value	0 (0.0)	1 (0.9)	> 0.9999
Increase creatinine (n.v. ≤ 0.7 mg/dL)			
≥ 1.5 x normal value	0 (0.0)	3 (2.6)	0.2577
≥ 2.5 x normal value	0 (0.0)	0 (0.0)	–
Increase urea (n.v. ≤ 46 mg/dL)			
≥ 1.5 x normal value	1 (0.9)	0 (0.0)	0.986
≥ 2.5 x normal value	0 (0.0)	1 (0.9)	> 0.9999
Decrease potassium (n.v. ≥ 3.4 mmol/L - 4.9 mmol/L)			
< 3.4 mmol/L	4 (3.6)	6 (5.2)	0.7897
< 2.4 mmol/L	0 (0.0)	0 (0.0)	–
Decrease calcium (n.v. ≥ 2.0 mmol/L - 2.6 mmol/L)			
< 2.0 mmol/L	12 (10.7)	7 (6.0)	0.2397
< 1.8 mmol/L	0 (0.0)	0 (0.0)	–
Decrease sodium (n.v. 134–145 mmol/L)			
< 134 mmol/L	14 (12.5)	23 (19.8)	0.1867
< 130 mmol/L	2 (1.8)	1 (0.9)	0.9756
Adverse reactions			
Exanthema	1 (0.9)	0 (0.0)	0.3302
Sweating	3 (2.7)	2 (1.7)	0.6228
Fever	5 (4.5)	10 (8.6)	0.2864
Loss of appetite	3 (2.7)	2 (1.7)	0.2057
Diarrhea	15 (13.4)	14 (12.1)	0.6228
Obstipation	4 (3.6)	3 (2.6)	0.7642

The occurrence of clinical and laboratory adverse reactions was not significantly different (*p* > 0.05) between both study groups

*Abbreviations*: *ALT alanine aminotransferase*, *AST aspartate aminotransferase, mg/dL* milligram per deciliter, *mmol/L* millimol per liter, *n* sample size, *n.v.* normal value, *No.* number, *U/L* Units per liter

Adverse events in the fosaprepitant group and the control group included exanthema, sweating, fever, loss of appetite, diarrhea, and obstipation. None of the patients in both study groups experienced neurological or anaphylactic adverse reactions or phlebitis of the fosaprepitant infusion site. The differences between the two groups were not statistically significant (*p* > 0.05; Table 3).

Fosaprepitant was administered during 17 (15.2%) of 112 courses of chemotherapy with ifosfamide within the first 3 months (November 2015 – January 2016) after the antiemetic prophylaxis regimen was changed to fosaprepitant. During these courses, we could not observe potentially drug-related neurological adverse events (Table 3).

## Discussion

Dexamethasone is prohibited in several pediatric chemotherapy protocols [5, 6]. Alternative or complementary antiemetic agents are needed for these patients. This non-interventional observation study is the first to report on the use of an antiemetic prophylaxis regimen consisting of single-dose intravenous fosaprepitant (NK_1_R antagonist) at a dosage of 4 mg/kg bodyweight in combination with ondansetron (5-HT_3_R antagonist) 24-h continuous infusion without dexamethasone in pediatric patients between 0.5 and 17.9 years of age receiving moderately or highly emetogenic chemotherapy.

The results show a favorable efficacy of fosaprepitant combination therapy when compared with antiemetic prophylaxis with ondansetron only. Adverse events were not significantly higher in the fosaprepitant group compared with the control group. The intravenous administration of fosaprepitant was well-tolerated. Clinical drug-related side effects were similarly low in both groups. New or different safety issues were not observed in this study compared with previous studies with fosaprepitant in adult and pediatric patients [3, 4, 7, 12, 13].

In a randomized double-blinded phase III clinical trial with a total of 302 pediatric patients with a median age of 7 years (0.5–17.8 years) receiving either oral aprepitant (up to 125 mg as capsule or suspension, depending on age) or a placebo combined with ondansetron on the first day of moderately to very highly emetogenic chemotherapy followed by 2 days with antiemetic prophylaxis with aprepitant (up to 80 mg) or placebo only, the number of patients with complete control of CINV (absence of vomiting) in the delayed phase was significantly higher under aprepitant (51%) compared with the placebo control group (26%). Hepatic or renal laboratory parameters or electrolytes were not assessed in this study [14].

In a retrospective chart analysis, 35 pediatric patients with a median age of 10 years (range 10 months - 18 years) who received 4 mg/kg fosaprepitant (max. 150 mg) in addition to an antiemetic prophylaxis regimen with ondansetron (100% of the patients), dexamethasone (69%), scopalamine (9%), dronabinol (6%), diphenhydramine (3%) and/or lorazepam (3%) for the prevention of CINV were analyzed. An absence of vomiting was observed in 89% of the patients during the acute and 63% in the delayed phase, and in 60% overall. In 12 of the 35 patients (34.2%), elevation of the transaminases AST and ALT occurred. The authors hypothesized that these increases were mainly caused by methotrexate as part of the chemotherapy regimen. Other hepatic or renal laboratory parameters were not analyzed in this study. The results were not compared with a control cohort without fosaprepitant [15].

In a randomized, double-blind, placebo-controlled phase III single-center trial, the efficacy, safety and feasibility of an antiemetic prophylaxis regimen with fosaprepitant, ondansetron and dexamethasone compared with ondansetron and dexamethasone and a placebo were investigated. A total of 163 pediatric patients between 1 and 12 years of age were prospectively enrolled. The placebo group (median age: 5 years) received an intravenous prophylaxis regimen with ondansetron bolus (1 × 0.15–0.3 mg/kg; max. 16 mg) plus dexamethasone (1 × 0.075 mg/kg) and normal saline (placebo) before chemotherapy and oral ondansetron (total dose 0.3 mg/kg per 8 h) and oral dexamethasone (total dose 0.15 mg/kg per 8 h) during the 48 h after the last administration of emetogenic chemotherapy. The therapy group received the same regimen but 3 mg/kg fosaprepitant in normal saline instead of the placebo. Complete absence of vomiting was significantly higher (*p* < 0.001) in the fosaprepitant group during the acute CINV phase (86% versus 60%), the delayed CINV phase (79% versus 51%) and both CINV phases (70% versus 41%) compared with the control group [4]. The distinctly worse results in our control group are most likely ascribed to the prophylaxis regimen without dexamethasone compared with the results of the study by Radhakrishnan et al., emphasizing the importance of dexamethasone as a crucial element in CINV prophylaxis. However, the comparison of the two studies’ results regarding their fosaprepitant groups gives rise to the question whether a double prophylaxis with fosaprepitant and ondansetron might be similarly effective as a triple prophylaxis with additional dexamethasone. This might be of great importance for pediatric patients receiving chemotherapy during which dexamethasone is prohibited. However, this hypothesis must be analyzed in future prospective trials.

## Conclusions

The data provided in this non-interventional observation study indicate that antiemetic prophylaxis with single-dose *intravenous* fosaprepitant in addition to a 24-h continuous infusion of ondansetron without dexamethasone was safe and effective as CINV prophylaxis in pediatric patients between 0.5 and 17.9 years of age receiving moderately or highly emetogenic chemotherapy. The prophylaxis regimen with fosaprepitant and ondansetron was significantly favorable compared with the mono-prophylaxis with ondansetron only in both the acute and the delayed CINV phase. Potentially drug-related adverse events of fosaprepitant could not be observed in this analysis. Larger prospective trials are necessary to evaluate our findings.

## Data Availability

The datasets used and/or analyzed during the current study are available from the corresponding author on reasonable request.

## Abbreviations

5-HT_3_R: 5-hydroxytryptamine-3 receptor
ALL: Acute lymphoblastic leukemia
AML: Acute myeloid leukemia
CG: Control group
CI: Confidence interval
CINV: Chemotherapy induced nausea and vomiting
FG: Fosaprepitant group
IV: Intravenous
L: Liter
MASCC/ESMO: Multinational Association of Supportive Care in Cancer/European Society for Medical Oncology
mg/dL: Milligram per deciliter
mg/m^2^: Milligram per square meter body surface area
mmol/L: Millimol per liter
*n*: Sample size
n.d.: Not determined
n.v.: Normal value
NCCN: US National Comprehensive Cancer Network
NK_1_R: Neurokinin-1 receptor
No.: Number
p: *P*-value, probability value
SD: Standard deviation
U/L: Units per liter
vs.: Versus

## References

[1] Navari RM (2017). Management of chemotherapy-induced nausea and vomiting in pediatric patients. Paediatric drugs.

[2] Aapro M, Carides A, Rapoport BL, Schmoll HJ, Zhang L, Warr D (2015). Aprepitant and fosaprepitant: a 10-year review of efficacy and safety. Oncologist.

[3] Okumura LM, D'Athayde Rodrigues F, Ferreira MAP, Moreira LB (2017). Aprepitant in pediatric patients using moderate and highly emetogenic protocols: a systematic review and meta-analyses of randomized controlled trials. Br J Clin Pharmacol.

[4] Radhakrishnan Venkatraman, Joshi Archit, Ramamoorthy Jaikumar, Rajaraman Swaminathan, Ganesan Prasanth, Ganesan Trivadi S., Dhanushkodi Manikandan, Sagar Tenali G. (2018). Intravenous fosaprepitant for the prevention of chemotherapy-induced vomiting in children: A double-blind, placebo-controlled, phase III randomized trial. Pediatric Blood & Cancer.

[5] Roila F, Molassiotis A, Herrstedt J, Aapro M, Gralla RJ, Bruera E (2016). 2016 MASCC and ESMO guideline update for the prevention of chemotherapy- and radiotherapy-induced nausea and vomiting and of nausea and vomiting in advanced cancer patients. Ann Oncol.

[6] Einhorn LH, Rapoport B, Navari RM, Herrstedt J, Brames MJ (2017). 2016 updated MASCC/ESMO consensus recommendations: prevention of nausea and vomiting following multiple-day chemotherapy, high-dose chemotherapy, and breakthrough nausea and vomiting. Support Care Cancer.

[7] Shillingburg A, Biondo L (2014). Aprepitant and fosaprepitant use in children and adolescents at an academic medical center. J Pediatr Pharmacol Therapeut.

[8] US Food and Drug Administration. EMEND - Fosaprepitant Dimeglumine. NDA 022023 - SUPPL-17 2018 [FDA Approval]. Available from: https://www.fda.gov/downloads/Drugs/DevelopmentApprovalProcess/DevelopmentResources/UCM605696.pdf. Accessed 1 May 2019.

[9] Dupuis LL, Boodhan S, Sung L, Portwine C, Hain R, McCarthy P (2011). Guideline for the classification of the acute emetogenic potential of antineoplastic medication in pediatric cancer patients. Pediatr Blood Cancer.

[10] U.S. NIH -NCI. Common Terminology Criteria for Adverse Events v4.03 (CTCAE) 2010. 2010 May 28, 2009.

[11] Fay Michael,P. (2010). Two-sided Exact Tests and Matching Confidence Intervals for Discrete Data. The R Journal.

[12] Grunberg S, Chua D, Maru A, Dinis J, DeVandry S, Boice JA (2011). Single-dose fosaprepitant for the prevention of chemotherapy-induced nausea and vomiting associated with cisplatin therapy: randomized, double-blind study protocol--EASE. J Clin Oncol.

[13] Saito H, Yoshizawa H, Yoshimori K, Katakami N, Katsumata N, Kawahara M (2013). Efficacy and safety of single-dose fosaprepitant in the prevention of chemotherapy-induced nausea and vomiting in patients receiving high-dose cisplatin: a multicentre, randomised, double-blind, placebo-controlled phase 3 trial. Ann Oncol.

[14] Kang HJ, Loftus S, Taylor A, DiCristina C, Green S, Zwaan CM (2015). Aprepitant for the prevention of chemotherapy-induced nausea and vomiting in children: a randomised, double-blind, phase 3 trial. Lancet Oncol.

[15] Timaeus S, Elder J, Franco K (2018). Evaluation of the use of Fosaprepitant for the prevention of chemotherapy-induced nausea and vomiting in pediatric patients. J Pediatr Hematol Oncol.

[16] Directive 2001/20/EC of the European Parliament and of the councol of 4 April 2001 on the approximation of the laws, regulations and administrative provisions of the Member States relating to the implementation of good clinical practice in the conduct of clinical trials on medical products for human use, 2001/20/EC. Sect. OJ L 121 (2001). 16276663

